# Endoluminal radiofrequency ablation of the main pancreatic duct is a secure and effective method to produce pancreatic atrophy and to achieve stump closure

**DOI:** 10.1038/s41598-019-42411-7

**Published:** 2019-04-11

**Authors:** Anna Andaluz, Elzbieta Ewertowska, Xavier Moll, Adrià Aguilar, Félix García, Dolors Fondevila, Rita Quesada, Enrique Berjano, Luís Grande, Fernando Burdío

**Affiliations:** 1 Departament de Medicina i Cirurgia Animals, Facultat de Veterinària, Universitat Autònoma de Barcelona, Barcelona, Spain; 2 BioMIT, Department of Electronic Engineering, Universitat Politècnica de València, Valencia, Spain; 3 Department of Surgery, Hospital del Mar, Barcelona, Spain

## Abstract

Radiofrequency energy has been used both experimentally and clinically to manage the pancreatic remnant after distal pancreatectomies. Our goal was to determine whether endoluminal radiofrequency (RF) ablation of the main pancreatic duct in large animals would be more efficient than glue occlusion as an exocrine pancreatic atrophy-inducing procedure. Thirty-four Landrace pigs were assigned to either the transpapilar (n = 16) or transection (n = 18) groups. The transection implied the pancreas neck was severed. In each of these groups the remaining distal pancreatic duct was occluded either by RF or by glue. In the transpapilar group complete atrophy was observed in all the RF cases, while atrophy was incomplete in all the members of the glue subgroup. The failure rate of the main pancreatic duct (usually expressed by a pseudocyst) in the transection groups was dramatically higher in the glue subgroup than the RF subgroups (9 out of 9 and 1 out of 9, respectively) and postoperative mortality occurred only in the glue subgroup (3 out of 9). These results show the superiority of endoluminal RF ablation over glue for main pancreatic duct occlusion, as seen by the degree of atrophy and fewer postoperative pancreatic fistulas.

## Introduction

In the history of the management of pancreatic diseases many attempts have been made to occlude the pancreatic ductal system. Since the pioneering experiments of insulin identification by Banting & Best in 1922, it has been well known that ligation/occlusion of the main pancreatic duct leads to complete exocrine atrophy with apparent preservation of the endocrine pancreas and without any significant signs of clinical pancreatitis^1^. This procedure has been extensively studied in the experimental setting as a model of expansion of β cell or mass-β-cell neogenesis and is also currently being studied as a novel therapy for diabetes^2–8^, usually by ligation of the pancreatic duct. Clinical studies involving intraductal injection of hardening glue have been carried out in two scenarios: (1) as a system of abolition of the pancreatic secretion and pain in chronic pancreatitis^9,10^, and (2) as a method of managing the pancreatic stump after pancreaticoduodenectomy (PD), usually after removal of the pancreatic tumor in the head of the pancreas^11–14^. However, this procedure has never found much acceptance by clinicians because of the high risk of failure in the form of recanalization of the main pancreatic duct (first indication) or fistula formation (second indication) which can affect more than 50% of the patients^15,16^. In spite of this, occlusion of the pancreatic duct (usually by glue or sutures) remains an option for management of the pancreatic stump after PD in “difficult circumstances”^16^. In fact, it is currently employed by some groups^12,13^ and highly recommended by others^17^.

Radiofrequency (RF) energy has been used both experimentally and clinically to manage the pancreatic remnant after distal pancreatectomies to seal the main and secondary pancreatic ducts^18,19^. Our group has also demonstrated in rat^20^ and porcine^21,22^ models that RF-based transection of the neck of the pancreas can safely seal the remnant of the pancreas and activate a massive exocrine atrophy with a lower risk of pancreatic fistula (from 75% to 14%, for suture and RF-based transection of the pancreas, respectively in the pig liver model). Interestingly, we have recently demonstrated in a Ptf1a-Cre(+/ki); K-ras LSLG12Vgeo(+/ki) mouse model that pancreatic duct ligation leads to fewer premalignant lesions in the ligated distal pancreas, which further justifies the search for an efficient method of pancreatic occlusion^23^.

RF energy has been extensively employed as a method of tumor ablation and has also recently been used as a system of vessel occlusion (for example in varicose veins). This effect is due to its unique property of shrinking the vessel wall and more specifically in achieving denaturation of collagen and other proteins by a controlled temperature rise with a bipolar RF system^24,25^, which can achieve complete occlusion of the vessels in 98.4% of the treated vessels^24^. Given the morphological similarity in the structure of the vessels and the pancreatic duct, we considered that an efficient endoluminal ablation system could achieve complete obstruction of the main pancreatic duct (MPD).

Our goal was therefore to determine whether endoluminal RF ablation of the main pancreatic duct would be more efficient than glue occlusion as an exocrine pancreatic atrophy-inducing procedure in a large animal.

## Material and Methods

### Animals

Thirty-four Landrace pigs with a mean preoperative weight of 34.7 kg (range 20–46 kg) obtained from the farm of the *Universitat Autònoma de Barcelona* (Spain) were used for the experimental procedures. All aspects of this study were regarded as part of an animal research protocol, in accordance with the guidelines approved by the Ethical Commission of the *Universitat Autònoma de Barcelona* (Authorization Number CEEAH 3487 and DMAH 9583) and the Government of Catalonia’s Animal Care Committee.

### Perioperative care

Preoperative care and anesthesia were provided by fully trained veterinary staff. All the animals were fasted for 12 hours before surgery. After initial sedation with a combination of azaperone and ketamine (4 and 10 mg/kg IM respectively), intravenous access was obtained through marginal ear-vein cannulation and analgesia was given previous to anesthesia (morphine 0.2 mg/kg IM and meloxicam 0.2 mg/kg IV). The anesthetic induction phase was performed with propofol (4 mg/kg IV). The trachea was intubated and anaesthesia maintained with 2–2.5% isoflurane (IsoVet, B. Braun) in 100% oxygen through a semi-closed circular anaesthetic system. Ventilation was controlled by intermittent positive pressure ventilator (SAV 2500, B. Braun) in order to maintain normocapnia during the entire anaesthetic period. All the animals received an infusion of lactated Ringer’s solution at a rate of 10 mL/kg/h during the perioperative period and IV antibiotic therapy (cephalexin, 20 mg/kg) was administered via the cephalic vein. Throughout the surgical process, temperature, cardiac frequency, respiratory frequency, capnography, arterial pressure, pulse and electrocardiography were monitored using a multifunction patient VetCare monitor.

### Groups and surgical procedure

The animals were assigned to either the transpapilar (TP, n = 16) or transection groups (TS, n = 18). Each of these groups was divided into two sealing method subgroups (radiofrequency (RF) and glue (G)). All the surgical procedures were performed by the same surgical team (AA and XM).

#### Transpapilar groups

**Figure 1:**
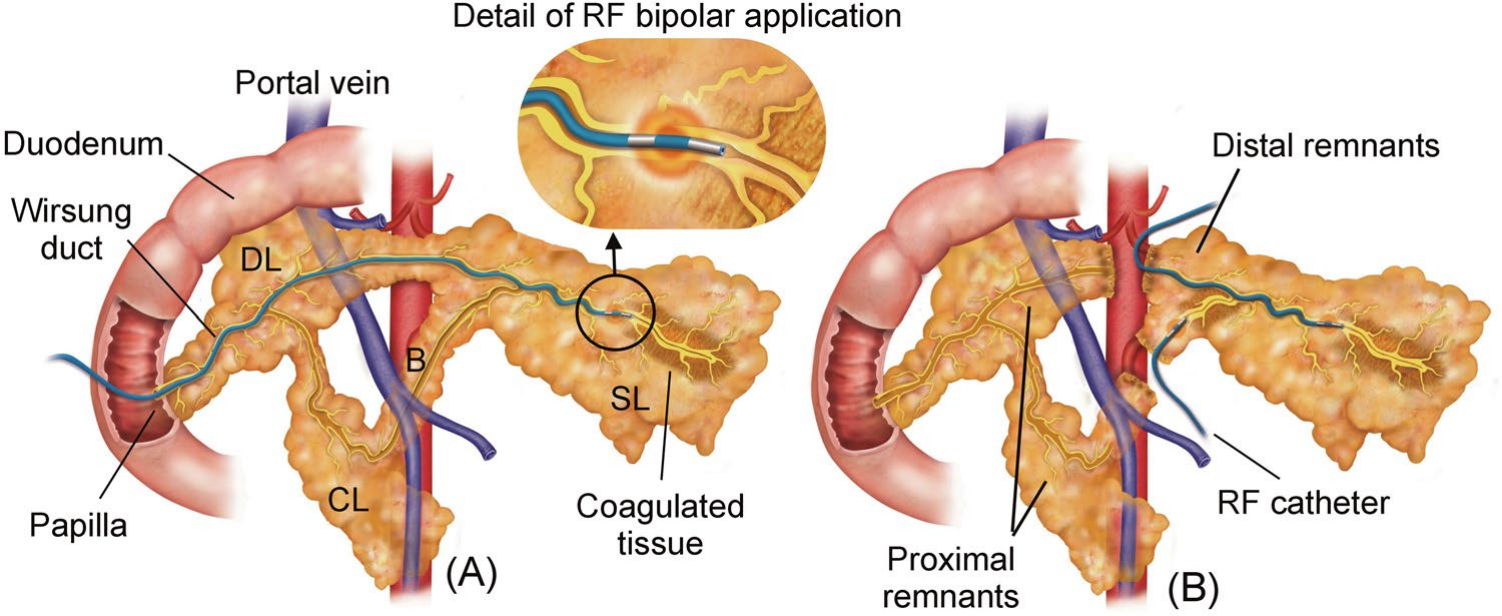
Surgical procedures conducted in the RF groups. (**A**) In the transpapilar group (TP-RF) a RF bipolar catheter was introduced through the papilla and gently pulled back while RF power was applied. (**B**) In the transection group (TS-RF), dissection of the pancreas neck over the portal vein was firstly performed, and the transection planes of the proximal remnants were coagulated and sealed with an internally cooled RF electrode. After that, the distal remnants were endoluminally treated with the same RF bipolar catheter employed used in the TP-RF group, and their transection planes coagulated and sealed with the internally cooled RF device. SL: splenic lobe; DL: duodenal lobe; CL: connecting lobe (CL); B: Bridge.

In the transpapilar group (TP) the Wirsung duct was occluded through the pancreatic papilla by open laparotomy and enterotomy. After locating the pancreatic papilla, two subgroups were formed: the transpapilar RF (TP-RF) and transpapilar glue group (TP-G). For the former group (n = 8) an RF bipolar sealing system was used. The catheter was introduced through the papilla and gently advanced into the Wirsung duct to achieve occlusion (see Fig. 1A). Information on the use of the device is given below. In the TP-G group (n = 8) the Wirsung duct was occluded with synthetic surgical glue (Glubran 2, Gem SRL, Viareggio, Italy) administered through a 4 Fr Jackson Cat catheter (Smiths-Medical, Dublin, OH, USA) introduced into the Wirsung duct via pancreatic papilla (see Fig. 1B), as described in Alfieri *et al*. (2018)^9^. During application the catheter was gently pulled back in order to fill the entire duct with glue. Enterotomy and laparotomy were closed in the conventional manner.

#### Transection groups

**Figure 2:**
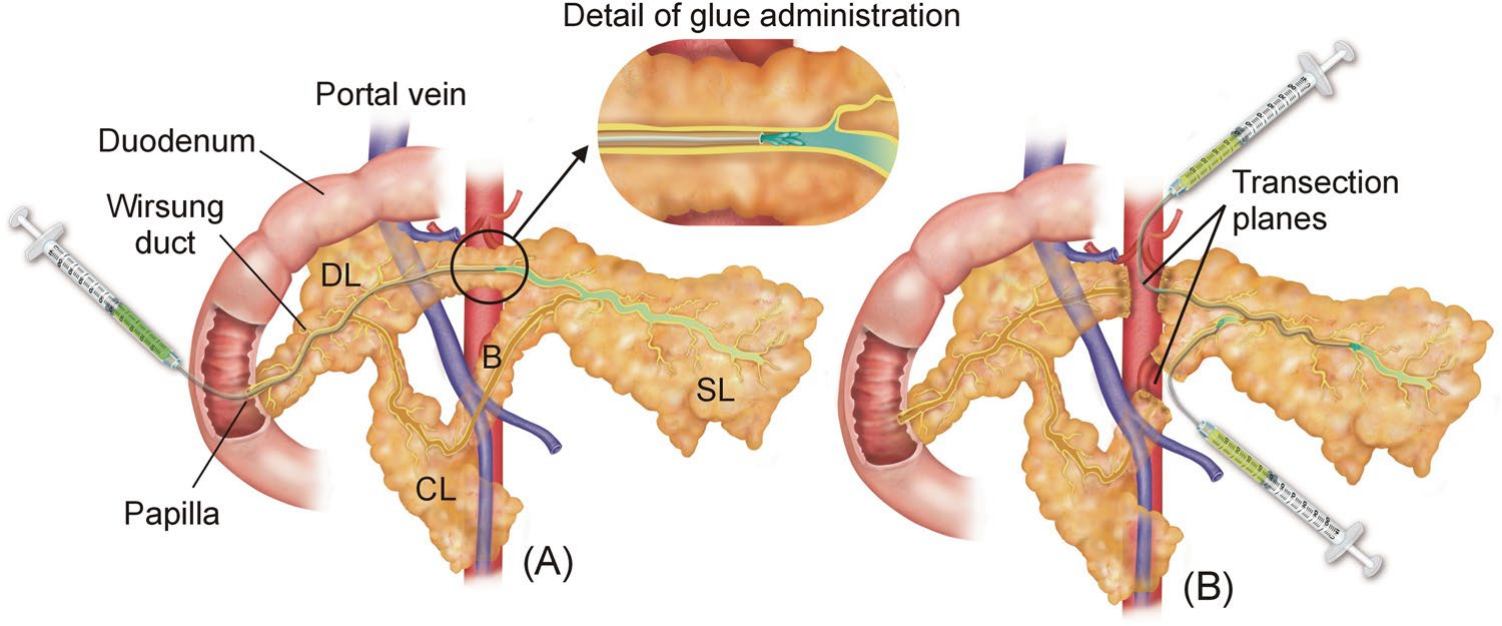
Surgical procedures conducted in the glue groups. (**A**) In the transpapilar group (TP-G) the Wirsung duct was occluded with surgical glue administered through a catheter which was introduced into the duct via pancreatic papilla, and gently pulled back as the glue completely fills the lumen. (**B**) In the transection group (TS-G) the pancreatic duct was first occluded by injecting glue through the catheter, and subsequently the remnants were sealed by applying an extra layer of glue. SL: splenic lobe; DL: duodenal lobe; CL: connecting lobe (CL); B: Bridge.

Dissection of the pancreas neck over the portal vein was performed via open laparotomy and the pancreas was transected by Mayo scissors. According to a previous study on pancreas anatomy of the pig^22^ the main pancreatic duct running along the body and tail of the pancreas coalesce with a common main pancreatic duct in the head by two ducts: one above the portal vein and another below this structure. This means that both ducts must be severed in order to achieve complete obstruction of the distal part (body and tail) of the pancreas (see Fig. 2). After transection, the proximal remnants were coagulated and sealed with a 3 mm diameter internally cooled RF electrode (Coolinside, Apeiron Medical, Valencia, Spain). The distal remnants were sealed differently in each subgroup. In the RF transection group (TS-RF) (n = 9) the bipolar device was first used to coagulate the Wirsung duct similarly to the intraluminal group (Fig. 2A). After ablation of the pancreatic duct, the pancreatic remnants were sealed with the internally cooled RF device. In the glue transection group (TS-G) the pancreatic duct was first occluded by injecting glue through the catheter (Fig. 2B). After obliteration of the pancreatic duct, the remnants were sealed by applying an extra layer of glue. A Blake Silicon Drain (Ethicon, Somerville, NJ, USA) was positioned adjacent to the pancreatic stump and extracted from the animal’s abdomen. The proximal end was subcutaneously tunneled to the animal’s back and connected to a reservoir. All wounds were closed in the standard fashion. The animals were allowed to awaken from anesthesia and were extubated when clinically indicated.

#### RF bipolar endoductal sealing system

The pancreatic duct was sealed by a 3 Fr two-pole catheter (Minitrode, Bioampere Research, Conselve, Italy), usually employed in clinical practice to apply pulsed RF for pain treatment. It has two metal electrodes (3 and 4 mm long) separated by 10 mm. It was connected to a model CC-1 RF generator (Radionics, Burlington, MA, USA) to create small circumscribed coagulation zones between both electrodes only (see Fig. 1C). In the first step of the ablation procedure the catheter was inserted as far as the distal end of the pancreatic duct in both the TP-RF and TS-RF groups. RF energy was applied between the electrodes at a constant voltage of 38 V while the catheter was progressively pulled back. When the proximal electrode was completely outside the duct, power application was stopped. The pullback technique was always performed manually by the same operator (AA), who monitored tissue impedance to ensure effective and safe dragging in terms of achieving an appreciable coagulation zone and avoiding overheating. The impedance value was displayed by the RF generator, which also provided an audible alert signal strictly related to impedance evolution during the ablation.

### Postoperative care

All the animals received water ad libitum for the first 24 h and subsequently were fed appropriate food twice daily. Antibiotics (amoxicillin 20 mg/kg IM, q24 h) were administered for the first three postoperative days. All the animals were inspected twice a day for the first seven postoperative days to detect any clinical signs of pancreatic leak or sepsis and to monitor debit and state of abdominal drains. They received morphine (0.1 mg/kg IM, q8h) for the first 16 postoperative hours and meloxicam (0.2 mg/kg IM, q24 h) for postoperative analgesia in the first five postoperative days. They were also given a 100 µg/h fentanyl patch for four days after the surgical procedure.

### Laboratory measurements

Peripheral blood was collected for measurement of serum amylase levels prior to the surgical procedure, 3 h after intervention, on days 2 and 7 postoperative (P.O.) and 4 weeks P.O before euthanasia. The blood samples were centrifuged at 2,500 × g for 10 min to extract the serum. Peripancreatic fluid amylase concentrations were measured from the drain tube (TS group) on days 3 and 7 P.O. and, if not present, during laparotomy 4 weeks P.O. The surgical drain was retired on day 7 P.O. Biochemical laboratory parameters were determined at the Centralized Analysis Center of the *Universidad Autònoma de Barcelona*, Veterinary Faculty, by technicians who were unaware of the study groups.

### Necropsy and histopathological study

Four weeks after pancreatic transection, all animals were again anesthetized, intubated, and ventilated as described above. Exploratory laparotomy was performed and the peritoneal cavity was assessed for excessive adhesions, free peritoneal fluid or any undrained collection/abscess. The pancreas (stump, uncinate process, and head) was dissected, removed, and temporarily placed in 10% buffered neutral formalin. The main pancreatic duct was identified and cannulated with an angiocatheter through both the ampulla in the duodenum and after cutting the pancreatic tail. The permeability of the duct (TP group) and the sealing of the stumps (in the TS group) was evaluated by injecting physiologic saline in an anterograde and retrograde manner. Thereafter, the specimen was immersed in 10% buffered neutral formalin for further histopathologic processing. The animals were then sacrificed with a commercial euthanasia solution.

Three-mm thick consecutive sections of the entire pancreas were taken from each animal. Two to four samples of the splenic lobe and connecting duodenal lobe were routinely processed and paraffin-embedded, cut to a thickness of 5 µm, stained with hematoxylin and eosin, and evaluated by light microscopic examination. The pathologist (DF), who was unaware of the experimental design, graded the severity of exocrine pancreas atrophy on a scale ranging from 0 to 5 known to maximize detection and repeatability^26^.

### Analyzed variables

In the TP group the primary outcome was the degree of atrophy of the exocrine pancreas. Secondary outcomes were: intraoperative complications, weight increase, alterations in stool consistency, extravasation of the physiologic saline during necropsy and other postoperative clinical parameters (anorexia, emesis, lethargy, and narcotic need).

In the transection group the primary outcome was the development of postoperative pancreatic fistula (POPF), defined as (1) macroscopic leak (evidence of dye extravasation from the pancreatic stump when catheterizing the distal main pancreatic duct), (2) any undrained amylase-rich fluid collections/abscess, or (3) greater than a threefold drain/serum amylase ratio after the third postoperative day, in accordance with the International Study Group of Pancreatic Fistula (ISGPF) guidelines^27^. Secondary outcomes were: intraoperative complications, weight increase, postoperative serum amylase concentration and peritoneal fluid amylase concentration, histopathologic alterations of the pancreatic remnant, and other postoperative clinical parameters (anorexia, emesis, lethargy, and narcotic need).

### Statistical analysis

Continuous variables are presented as median and minimum-maximum value. The Kolmogorov–Smirnov test was used to determine the variables’ distribution. Student’s t test was used to make pairwise comparisons of normal distributed parameters, and the Mann-Whitney U test was used for nonparametric data. Dichotomous variables were compared using the Chi-square test. The laboratory analyses that included repeated measures were evaluated by two-way analysis of variance with the Bonferroni test for posthoc anaylisis. Data collection and analyses were performed with Statistical Package for the Social Sciences (Version 19.0, SPSS Inc, Chicago, IL, USA).

## Results

### Intraoperative features

There were no intraoperative deaths or major complications during surgery. However, one animal in TP-RF group was excluded from the study due to failure of the RF generator during ablation. For this reason, 15 animals were finally included in the transpapilar group, 7 in the TP-RF and 8 in the TP-G group. Due to a breakdown of the RF bipolar device, the ablation technique was considered incomplete in animal TS-RF-6.

### Postoperative follow-up

**Figure 3:**
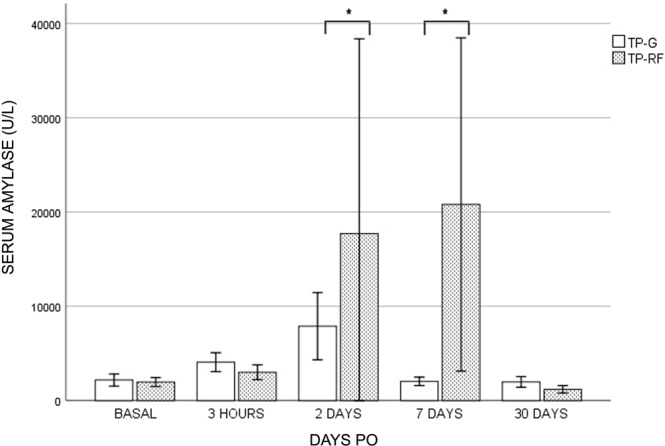
Serum amylase levels in transpapilar group (TP-G and TP-RF) during postoperative. Note significant differences (p < 0.05) in serum amylase levels on days 2 and 7 PO (*).

The study variables in the TP and TS groups are shown in Tables 1 and 2, respectively. About TP group, all the animals were killed on day 30 PO. No animals died or suffered serious alterations during the postoperative period. Significant differences (p < 0.05) were observed in weight increase, being greater in the TP-G group. All the animals in the TP-RF group presented alterations of stool consistency, ranging from totally liquid to pasty, during the entire postoperative period. Four animals in the TP-RF group required pancreas enzyme substitution. Those in TP-G presented completely normal stools without the need for enzymatic supplementation. The TP-RF animals presented statistical differences (p < 0.05) in serum amylase levels on days 2 and 7 PO (Fig. 3).

**Table 1:** Study variables in transpapilar groups (TP).

Group	Animal	Weight Gain (kg)	Stool Consistency	Duct Patency	Score of Atrophy (0–5)		
					Proximal	Distal	Uncinate
TP**-**RF	**1**	2.6	Diarrhea (soft)	No	5	5	5
	**2**	0.7	Normal	No	5	5	5
	**3**	0.6	Diarrhea (soft)	No	5	5	5
	**4**	9.4	Diarrhea (soft- liquid)	No	5	5	5
	**5**	0.7	Diarrhea (soft- liquid)	No	5	5	4
	**6**	0.4	Diarrhea (soft- liquid)	No	3–4	5	4
	**7**	3.9	Diarrhea (soft- liquid)	No	4	5	5
	**Mean ± SD**	**2.6 ± 3.3**					
P value**		**0.001**	**0.0002**	**0.0002**	**0.004**	**0.001**	**0.04**
TP-G	**1**	9	Normal	Yes	0	5	0/5*
	**2**	7.2	Normal	Yes	0/5*	0/5*	0/5*
	**3**	4.4	Normal	Yes	0/5*	0	5
	**4**	11.2	Normal	Yes	0	0	0/5*
	**5**	11	Normal	Yes	0	0	0/2–3/5*
	**6**	11.7	Normal	Yes	0/4*	0/5*	0/5*
	**7**	17.3	Normal	Yes	0	0	0/5*
	**8**	11.8	Normal	Yes	0	0	5
	**Mean** ± **SD**	**10.5** ± **3.8**					

*Non-homogeneous atrophy pattern (lobular atrophy).

**For comparisons between both groups (TP-RF vs TP-G).

**Table 2:** Study variables in transection group (TS).

Group	Animal	Weight Gain (kg)	Duct Patency	>Threefold Drain/Serum Amylase > 3 po	Pseudocyst	Mortality	Score of Atrophy (0–5)		
							Proximal	Distal	Uncinate
TS-RF	**1**	18.6	No	No	No	**No**	0	5	4–5
	**2**	5.1	No	No	No	**No**	5	5	5
	**3**	16.6	No	Yes	No	**No**	0	5	5
	**4**	18.2	No	No	No	**No**	0	5	5
	**5**	12.8	No	Yes	No	**No**	0	5	5
	**6**	15.8	No	**No**	Yes	**No**	0	5	5
	**7**	6.2	No	No	No	**No**	0	5	5
	**8**	5	No	No	No	**No**	0	5	5
	**9**	12.5	No	No	No	**No**	0	5	5
	**Mean** ± **SD**	**12.3** ± **5.6**							
P value **		**0.063**	**0.0001**	**0.49**	**0.008**	**0.058**	**NS**	**NS**	**NS**
TS-G	**1**	13.3	Yes	No	Yes	**No**	0	5	5
	**2**	—	Yes	—	—	**Yes**	—	—	—
	**3**	11.1	Yes	No	Yes	**No**	0	5	4–5
	**4**	12.3	Yes	No	Yes	**No**	0	5	5
	**5**	8.4	Yes	No	Yes	**No**	0	0/5*	5
	**6**	6.3	Yes	No	Yes	**No**	0	5	5
	**7**	—	Yes	Yes	Yes	**Yes**	0	5	5
	**8**	12.5	Yes	Yes	Yes	**No**	0	4–5	5
	**9**	—	Yes	Yes	Yes	**Yes**	0	2–3	2
	**Mean** ± **SD**	**6.7** ± **6.1**							

*Non-homogeneous atrophy pattern (lobular atrophy).

**For comparisons between both groups (TS-RF vs TS-G).

**Figure 4:**
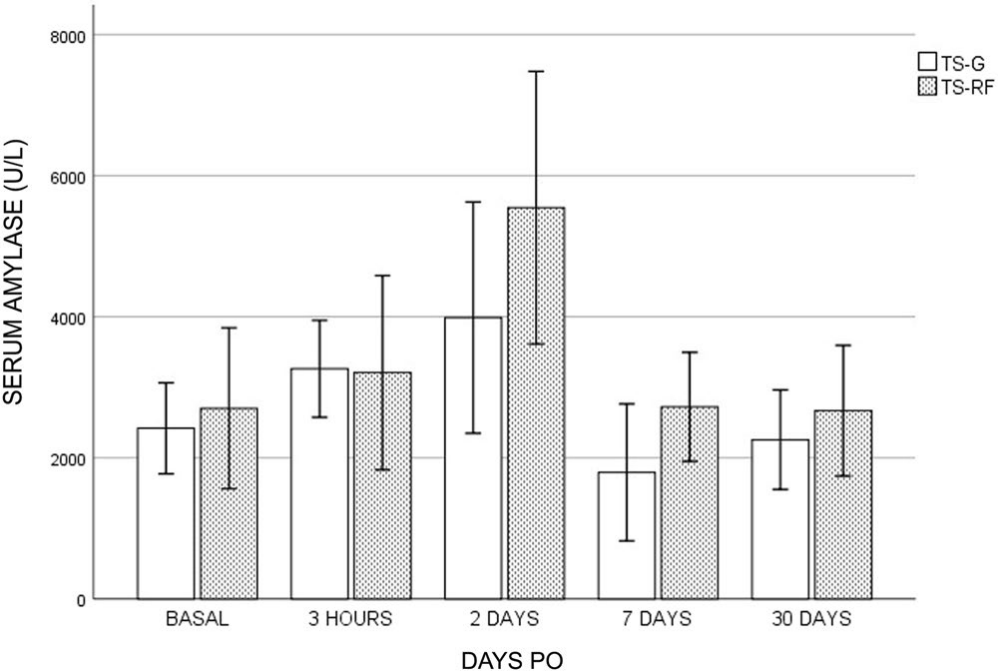
Serum amylase levels in transection group (TS-G and TS-RF) during postoperative. In this case no significant differences among groups were found.

About the TS group, three animals died during the postoperative period. Pig TS-G-2 presented apathy and anorexia from day 3 PO. Due to its poor condition and lack of improvement, it was decided to euthanize it on day 5 PO. At necropsy, total duodenal obstruction with gastric dilatation was observed due to strong adhesions around the duodenum produced by the glue. Pig TS-G-7 evolved correctly until day 12 PO, at which time it began to be extremely apathetic and showed abdominal distension. Abdominal ultrasound showed pronounced gastric distension and the presence of an anechoid structure, about 10 cm in diameter caudal to the stomach, compatible with a pseudocyst. Due to lack of recovery it was sacrificed on day 13 PO. At necropsy, a large number of adhesions were seen to cause total duodenal obstruction between the cyst and various intestinal loops. Subject TS-G-9 died spontaneously on day 8 PO. Until then it had been slightly apathetic with sporadic vomiting. At necropsy, multiple adhesions were seen between the intestinal loops without any obstructions. A pseudocyst about 4 cm in diameter was located in the transection area. Analysis of the pseudoquist and abdominal fluid showed a high amylase content. In these three animals a leak was also found when the main pancreatic duct was injected with physiologic saline in the distal pancreas and extravasated at the same level in the pancreatic stump. The remaining animals were killed on day 30 PO. Of these animals, one in the TS-RF and one in the TS-G group presented slight postoperative complications consisting of apathy and anorexia, which evolved correctly with supportive treatment of analgesia and fluid therapy. No significant differences in weight gain and plasma amylase levels (Fig. 4) were found between the animals in the TS group.

### Pancreas atrophy and duct patency in transpapilar groups

**Figure 5:**
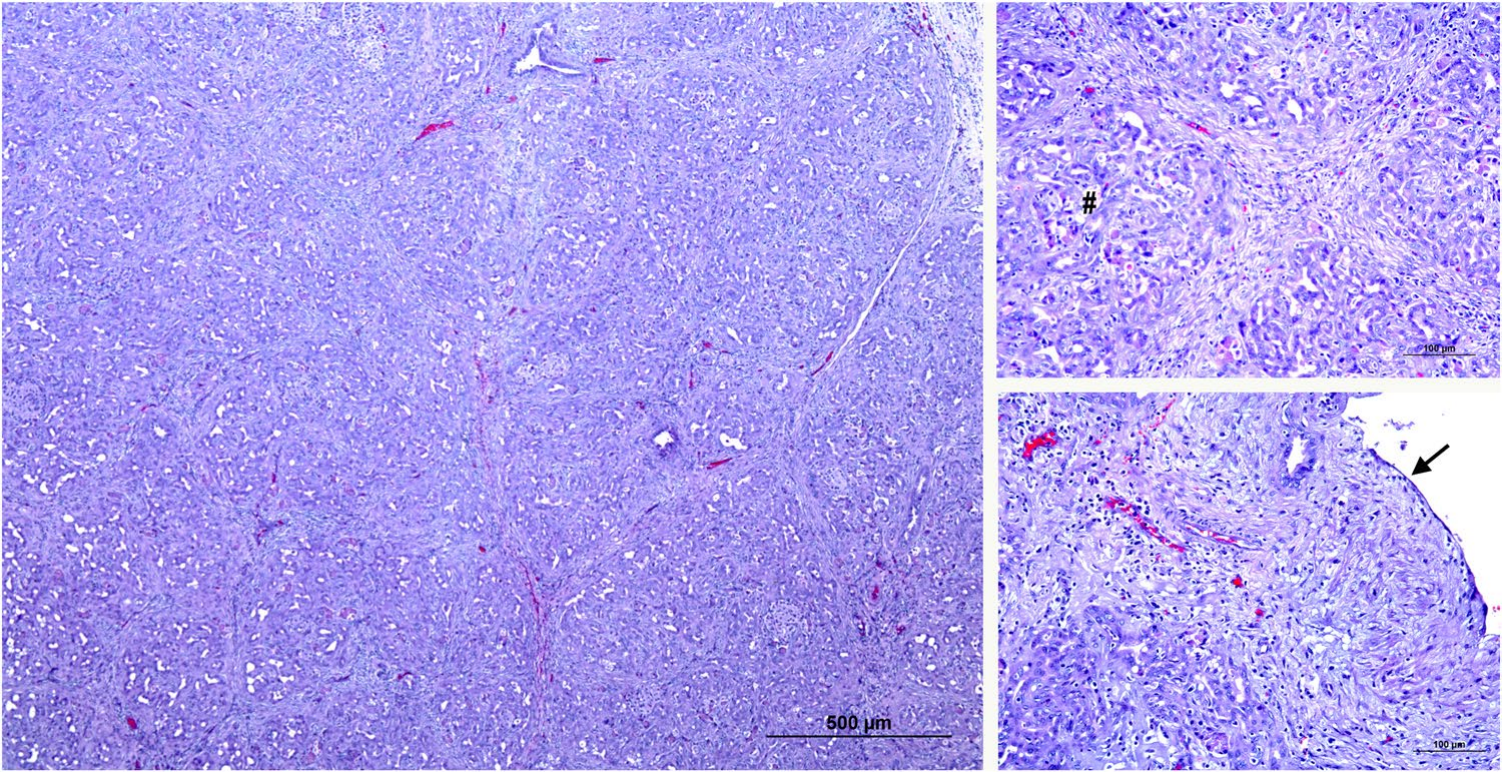
Microscopic pattern found in the pancreas of the TP-RF group (**A**–**C**). Common histological features of complete atrophy. The acinar component of the pancreatic tissue has completely disappeared (atrophy score 5) (#). The main pancreatic duct (black arrow) has lost its typical epithelium.

**Figure 6:**
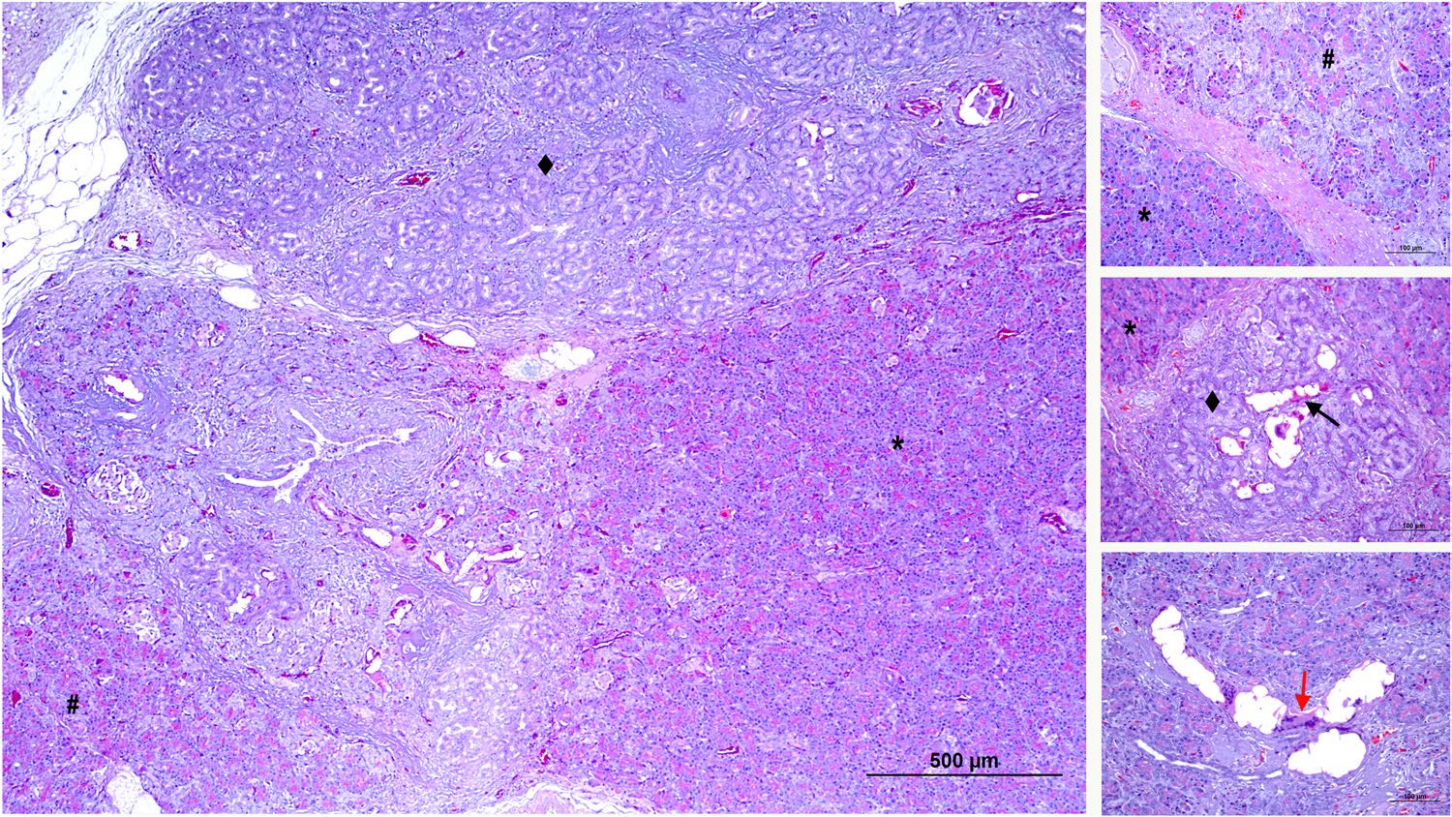
Microscopic pattern found in the pancreas of the TP-G group (**A**–**C**). In this group the loss of the acinar component showed a lobular pattern, with lobules without atrophy (score 0) (*), lobules with irregular and moderate atrophy (score 3) (#), and others with complete disappearance of exocrine pancreatic tissue (score 5) (◆). The glue can be seen inside the ducts of the atrophied lobes (black arrow). In these ducts surrounded by multinucleated cells there is also an obvious foreign body reaction (red arrow).

There were significant differences in duct patency between the groups (p < 0.05). All the animals of the TP-RF group had complete occlusion of the main pancreatic duct along the ablation area, while all those in group TP-G presented permeable ducts at the time of necropsy. Different atrophy patterns were observed in groups TP-RF and TP-G. The former group shows a homogeneous atrophy with almost complete and widespread disappearance of the acinar component (atrophy score 5) (Fig. 5). In the latter the loss of the acinar component showed a lobular pattern, with lobules without atrophy (score 0), with irregular and moderate atrophy (score 3), and others in which the exocrine pancreatic tissue had completely disappeared (score 5) (Fig. 6). In the TP-G group, glue was found inside the ducts of the atrophied lobes, in which there was also an obvious foreign body reaction, with the presence of multinucleated cells.

### Postoperative pancreatic fistula (POPF) and associated histological features in transection group

There were significant differences in duct patency (p < 0.05). All the animals in the TS-G group leaked through the transection area after canalization of the main pancreatic duct in the tail and physiological saline infusion. In all these cases, a connection was always found between the pseudocyst and the main duct from the distal pancreatic remnant. None of the animals in TS-RF showed leaks in the transection area after the injection of physiologic saline through the main pancreatic duct.

Likewise, significant differences were observed in the presence of pseudocysts between the two groups (P < 0.05). In TS-RF, only one pseudocyst was found in animal TS-RF-6, in which ablation was considered incomplete due to a breakdown of the bipolar RF device during the procedure, while in TS-G all the animals presented different sizes of pseudocyst.

**Figure 7:**
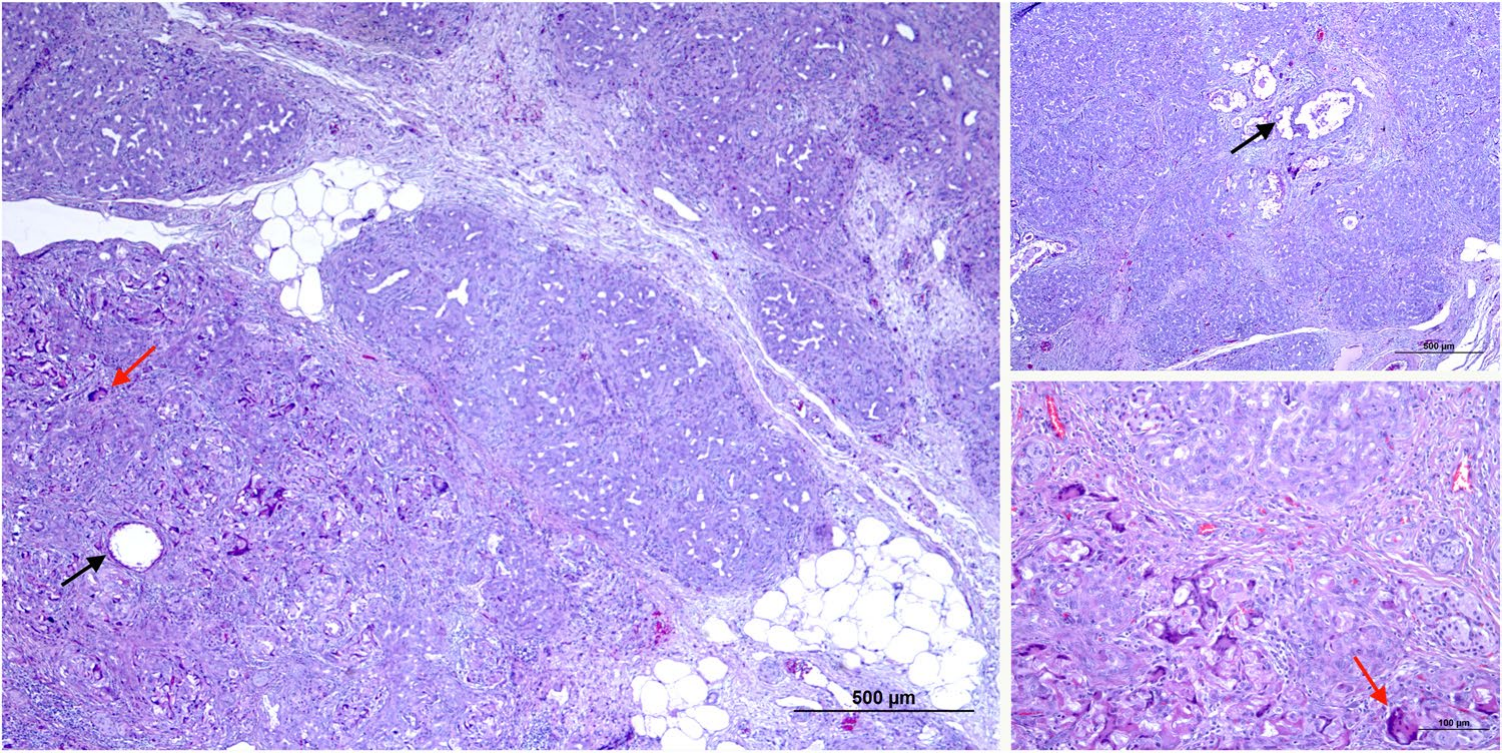
Microscopic pattern found in the pancreas of the TS-G group (**A**–**C**). The glue can be seen inside the ducts of the atrophied lobes (black arrow). In these ducts surrounded by multinucleated cells there is also an obvious foreign body reaction (red arrow).

No significant POPF differences were observed. Two animals in TS-RF and three in TS-G presented a greater than threefold drain/ serum amylase ratio after day 2 PO. No microscopic changes were observed in sections of the proximal pancreas samples, with complete microscopic preservation of the pancreatic architecture in all cases. Only one animal in TS-RF showed a complete atrophy of the proximal pancreas. In the distal sample sections, the acinar component had completed disappeared (atrophy score 5) in most of the animals in both groups. In TS-G the glue was visible inside the ducts of the atrophied lobes, in which there was also an obvious foreign body reaction with the presence of multinucleated cells (Fig. 7).

## Discussion

In spite of the initial high PD mortality rates, since the 1990s large retrospective series from specialized centers around the world have set a postoperative mortality benchmark of less than 5%^28,29^, although its morbidity high (over 40%). The main contributing factor is POPF, involving leakage of pancreatic juice from the pancreatic anastomosis, which can lead to severe secondary complications such as intra-abdominal abscesses and erosion bleeding^29,30^. In fact, in a recent multicenter randomized study comparing pancreatogastrostomy and pancreatoyeyunostomy in 14 high-volume German academic pancreatic surgery centers, the overall incidence of grade B/C fistula (clinically relevant POPF) was 21%, mainly in the soft pancreas. This study also honestly acknowledged that POPF is still substantial and underestimated, which could mean the actual postoperative mortality is over the 5% margin, even in high-volume academic pancreatic surgery centers. Similar conclusions were reached in a French multicenter randomized trial which found 42% POPF and main pancreatic diameter <3 mm in the soft pancreas subgroup, regardless of pancreatic anastomosis type (to jejunum or stomach)^31^.

It therefore seems there is a lot of room for improvement in this normal pancreas subgroup. However, occlusion/ligation of the main pancreatic duct in the commonly used glue occlusion approach is not usually any better than pancreatic anastomosis at managing the pancreatic stump after PD. In fact, in the last prospective randomized trial on PD glue occlusion versus pancreaticojejunostomy, similar complication rates were found despite similar exocrine insufficiency rates. These complications are similar to those described after the use of other sealing methods, such mechanical staples or conventional ligatures^22^. It was also suggested there might be a possibly higher risk of diabetes in the occluded pancreatic duct group, even though this study was not able to correctly evaluate this variable^11^.

Although pancreatic duct ligation has in fact been extensively studied in the experimental setting as a model of expansion of β–cell mass –β-cell neogenesis–^2–8^, it has always been done in small animal models, where ligation of the main pancreatic duct is simple and with no risk of fistulas^21^. However, ligation of the larger main pancreatic duct in humans is not better; Reisman *et al*.^32^ found POPF in 94% of patients in a controlled study after PD and ligation of the main pancreatic duct.

In the present study, we compared the use of surgical glue with the thermal ablation produced by a bipolar endoluminal RF device, both applied endoluminally, with two purposes. Firstly, to evaluate the degree of atrophy achieved with each of the methods, and secondly to compare the rate of pancreatic fistula after the section of the pancreas. According to the authors’ knowledge, there are no previous data regarding the endoluminal RF application neither in pancreatic surgery nor in any other minimally invasive procedure. RF applied on the surface of the stump after pancreatectomy has been tested in different studies^19,21,22^ showing to be a superior method, in terms of decreasing pancreatic fistulas when compared with staplers or surgical glue^33^. It should be noted, that surgical glue was chosen for the control group since it is applied endoluminally, as the bipolar RF device presented here. Therefore, among the sealing methods described in the literature, it would be the one that shares the most similarities with our endoluminal RF device.

In the present study on a large animal model we also found a high risk of failure when glue was used to occlude the main duct. After anterograde and retrograde infusion of physiologic saline, duct patency was found in all the animals of both the transpapilar and transection glue groups at autopsy on day 30 PO. This almost systematic failure of main pancreatic duct occlusion was clearly linked to pseudocyst formation in all the animals in the TS-G group.

Not surprisingly, irregular patterns of histology preservation were described in the occluded pancreas in the TP-G group, which likely account for the normal stool consistency and weight gain in these animals. The final clinical repercussion of this failure in the TS-G group was also a high mortality rate (3 of 9 animals). The absence of increased peritoneal amylase in many of the animals in the TS-G group could have been due to the difficulty of obtaining samples through drainage. Necropsy revealed that the TS-G group had a higher degree of peritoneal adhesions and fibrin than those in the TS- RF group, which hindered the recovery of drained fluid. In addition, there could have been fluid rich in pancreatic amylase inside the pseudocysts, which would not have been able to exit through the drain.

In sharp contradiction to the glue groups, in both groups in which endoluminal RF ablation of the main pancreatic duct was performed, neither duct patency nor mortality was found. The efficient occlusion of the main duct led to almost complete atrophy of the occluded pancreas in the TS-RF and TP-RF groups. Further clinical proof of pancreatic atrophy was given by the soft consistency of all the animals’ stools and the significant weight loss in the TP-RF group, similar to that described by other authors^34–36^ (in the TS-RF group, preservation of the proximal pancreas accounts for the insignificantly different weight gain over the TS-G group). Although some of the TS-RF animals presented more than threefold drain/serum amylases, it should be noted that these cases were asymptomatic and did not present pseudocysts. In addition, complete closure of the major pancreatic duct was observed in all the animals during necropsy. The only pseudocyst found in this group was directly associated with the rupture of the bipolar probe during the procedure. In this subject, the pancreatic remnant was sealed only by applying monopolar RF in the transection area, a technique that is only associated with 14% POPF incidence^22^. The increase in peritoneal amylases in the TS-RF group could be explained by the RF-induced cytolysis effect, as has previously been described^19^.

Why then is the behavior of the main pancreatic duct so different with glue and thermal ablation? No previous histological studies could give us a consistent answer, simply because no similar studies are available in prevention or treatment of POPF. Only some vaguely similar studies on endovascular RF ablation of varicose veins have demonstrated shrinkage and scarred vein trunks^24,25^. In the present study, glue could almost always be found inside the main pancreatic duct in the histological study at autopsy, usually unstuck from most of the epithelium, possibly because of epithelium regeneration, accounting for the irregular occlusion patterns. However, duct ablation led to necrosis of the epithelium and fibrosis of the intima, with the associated shrinkage and occlusion of the main duct. If there is no functional exocrine duct, no POPF can be expected.

Certain limitations of this study have to be acknowledged. First, large animals were involved with similar pancreas size to humans, in which the risk of POPF is radically different to small animals^21^, in spite of the remaining anatomical differences (see Figs 1 or 2). Second, hardening of the glue during application in the duct system may have impeded subsequent glue application^37^. Even if this is acknowledged as a shortcoming of this method, a valuable lesson has been learned. And third, to our knowledge, this is the first study to consider endoluminal thermal ablation of an exocrine gland duct. Further studies should confirm these encouraging results.

In view of the results obtained it can therefore be concluded that endoluminal RF-bipolar ablation is a safer and more efficient method than glue occlusion as an exocrine pancreatic atrophy-inducing procedure.
